# Efficacy of the spring distraction system for different etiologies of early onset scoliosis: evaluating an evolving treatment concept

**DOI:** 10.1007/s43390-026-01368-0

**Published:** 2026-06-02

**Authors:** Casper S. Tabeling, Isabelle E. Blaauw, Hilde W. Stempels, Tom P. C. Schlösser, Keita Ito, René M. Castelein, Moyo C. Kruyt, Justin V. C. Lemans

**Affiliations:** 1 Department of Orthopedic Surgery, University Medical Center Utrecht, P.O. Box 85500, 3508 GA Utrecht, The Netherlands; 2 Department of Biomedical Engineering, Eindhoven University of Technology, Eindhoven, The Netherlands; 3 Department of Developmental BioEngineering, University of Twente, Enschede, The Netherlands

**Keywords:** Early onset scoliosis, EOS, Spring, Spring distraction system, SDS, Growth-friendly, Etiology

## Abstract

**Purpose:**

Early onset scoliosis (EOS) is a challenging condition that often requires “growth-friendly” implants to control the curve and accommodate spinal growth, especially in young patients with severe curves. The spring distraction system (SDS) was developed to support growth without the need for repeated lengthenings. This prospective cohort study aimed to compare the performance of the SDS between patients with different EOS etiologies.

**Methods:**

We analyzed all SDS patients with a minimum of two-year follow-up. We measured curve correction, sagittal parameters, spinal growth, complications and unplanned return to the OR (UPROR) and compared these outcomes between different etiological groups. Differences between pre- and postoperative were compared with a mixed repeated-measures ANOVA. Differences between postoperative and later follow-up were compared using linear mixed models. Complications and UPROR rates were compared with a Kaplan–Meier survival analysis.

**Results:**

Sixty-four patients were included (14 congenital, 41 neuromuscular, and 9 idiopathic). Mean age at surgery was 8.4 ± 1.7 years. Follow-up was 3.7 ± 1.4 years. Correction was maintained for congenital and neuromuscular patients. In idiopathic patients, Cobb angle increased during follow-up (3.6°/year). T1-S1 growth was 7.4 mm/year (idiopathic), 8.9 mm/year (congenital), and 9.8 mm/year (neuromuscular). Fifty-nine complications occurred (mostly implant-related), corresponding to a complication rate of 0.22/patient/year (congenital), 0.26/patient/year (neuromuscular) and 0.27/patient/year (idiopathic). Forty-two UPRORs were recorded, corresponding to an UPROR rate of 0.19/patient/year (congenital), 0.20/patient/year (neuromuscular) and 0.07/patient/year (idiopathic).

**Conclusion:**

The SDS performs well across EOS etiologies in terms of curve correction, spinal growth, and complication/UPROR rates. Idiopathic patients had less sustained correction. Complication rates were comparable, idiopathic patients had the lowest UPROR rate.

**Trial registration:**

GRADS study (NL55705.041.16).

BiPOWR study (NCT04021784).

**Supplementary Information:**

The online version contains supplementary material available at https://doi.org/10.1007/s43390-026-01368-0.

## Introduction

Progressive early onset scoliosis (EOS) may result in cardiopulmonary compromise and loss of health-related quality of life (HRQoL) [1]. Various etiologies are relevant as described in the C-EOS classification: congenital/structural, neuromuscular, syndromic and idiopathic [2]. Currently, treatment for EOS patients is mostly guided by age and curve size, although each etiology has typical spinal characteristics that likely require tailored treatment strategies [3]. The aim of treatment is to control the deformity, while allowing growth for optimal pulmonary function [4]. When conservative management fails, surgical intervention is indicated [5–9]. Several “growth-friendly” implants have been developed, including the traditional growing rod (TGR), and the magnetically controlled growing rod (MCGR). While these techniques are effective, they have disadvantages that burden the patient and limit their efficacy [10–14].

The most important issue with these rigid, distraction-based implants is the need for periodic surgical or outpatient lengthenings using relatively high and abrupt forces (> 250N). This not only poses a burden to the patient, but also risks damage and subsequent stiffening of the spine, which may result in diminishing returns [15–18]. Additionally, high complication- and failure rates are associated with the complex distraction mechanisms (e.g., MCGR) and repetitive surgeries and anaesthesia.

To address these limitations, the spring distraction system (SDS) was developed as a continuous and dynamic distraction system [19, 20]. This system exerts a continuous distraction force via a compressed helical spring around standard growing rods that slide through a side-to-side connector. The device was developed in 2015, to treat several exceptional EOS cases, but its indications for use were broadened to include most EOS patients with a progressive curve that cannot be controlled conservatively [19]. Due to the versatility of the system, different strategies are used for different types of scoliosis, for example long bipolar fixation with bilateral springs for neuromuscular cases, and short hybrid fixation with unilateral spring distraction combined with a contralateral sliding rod for idiopathic cases. The implant is not yet registered; therefore, all patients are meticulously followed up in prospective clinical trials. Two-year follow-up data have shown promising results, including adequate curve correction and -control, physiological levels of spinal growth, and improved HRQoL [17, 20, 21]. However, whether SDS treatment demonstrates similar efficacy and safety across different EOS etiologies remains unknown. The current study aims to investigate the performance of SDS as used for different etiologies.

## Materials and methods

### Study design and inclusions

We included all patients that were treated primarily with SDS (no conversions from another system) that participate in the prospective clinical studies: GRADS (Research Portal number NL55705.041.16) and BiPOWR (Clinicaltrials.gov ID: NCT04021784 RP number NL64018.041.17) with at least two years of follow-up. Before SDS treatment, conservative treatments were tried, but unsuccessful. Exclusion criteria for SDS were connective tissue diseases (e.g., Marfan Syndrome) or severe bone pathology (e.g., osteogenesis imperfecta). When a patient graduated from SDS treatment (defined as either undergoing final fusion or reaching sufficient skeletal maturity with SDS in situ, whichever comes first), the measurements were performed on the radiograph before graduation (thus always with the SDS in situ).

### Spring distraction system

**Fig. 1:**
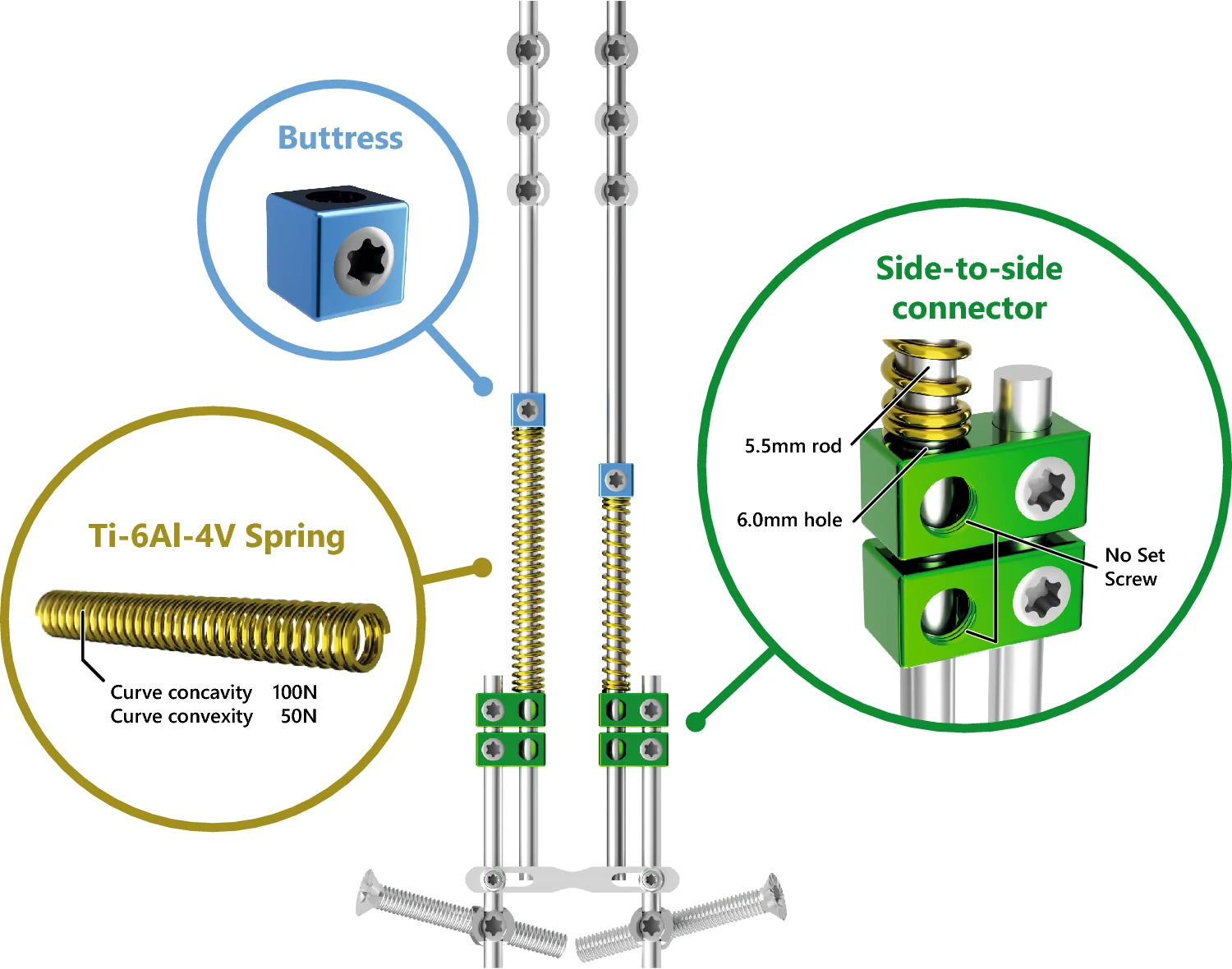
The Spring Distraction System (SDS): The SDS has three components added to standard CoCr growing rods (4.5 or 5.5 mm) to offer a continuous distraction force. The configuration generally used in neuromuscular EOS patients is shown, with ilio-sacral screws as the distal anchor instead of pedicle screws. **Green:** Two stacked side-to-side connectors with an oversized hole for the sliding rod. **Gold:** Ti6Al4V springs ranging from 50N to 150N. **Blue:** Buttress to tension the spring against the connector

**Fig. 2:**
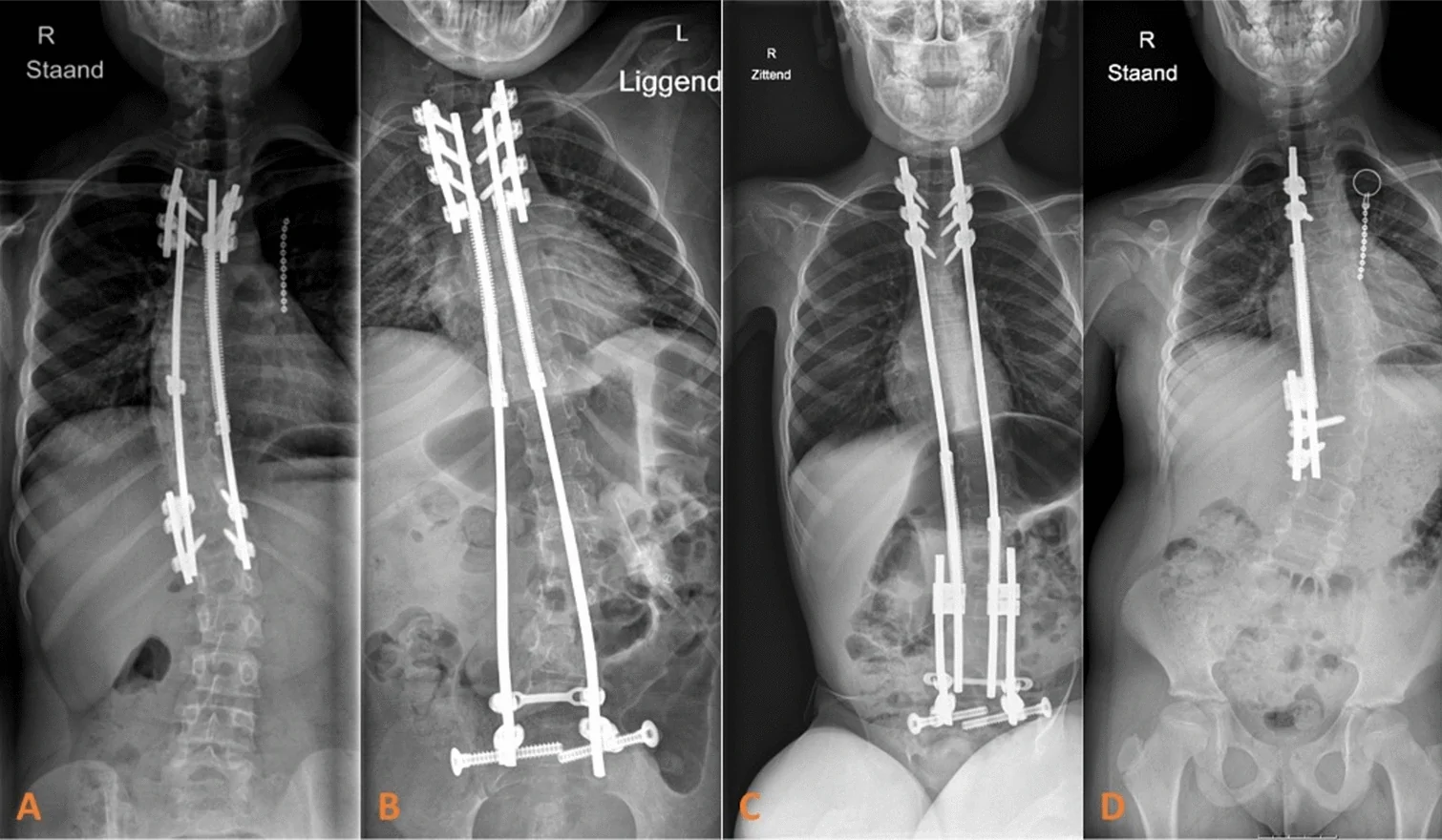
SDS configurations: **A** Hybrid construct with a unilateral SDS and a contralateral passive sliding rod fixed to the apex, typically used for idiopathic EOS. **B** Bilateral construct with two similar springs (bilateral 75N springs) and **C** Bilateral construct with two different spring forces (concavity: 100N, convexity: 50N). **D** Unilateral construct typically only used for congenital deformities

The SDS consists of one or more titanium (Ti-6Al-4V) helical coil springs positioned around a standard cobalt-chrome (CoCr) sliding rod (Fig. 1). The spring exerts pressure against a side-to-side connector that is left unlocked on the CoCr sliding rod, allowing continuous distraction. The spring is pre-tensioned using a standard buttress (Stryker, Leesburg, VI, USA) to provide a continuous distractive force which linearly declines with lengthening according to Hooke’s law (Fs = k ∙ x). The springs can be positioned strategically either uni- or bilaterally and with different forces, as shown in Fig. 2. When a spring has fully expanded (usually 4-6 cm) before end of growth, it can be re-tensioned with a minor surgery, similar in scope to a TGR lengthening surgery. Different strategies were employed for different types of scoliosis: congenital deformities were typically treated with unilateral convex hemi-epiphysiodesis combined with a concave distraction; idiopathic types with hybrid systems consisting of concave distraction and a convex sliding rod [22, 23] and neuromuscular patients with bilateral spring distraction.

Insertion is similar to other distraction devices via two separate incisions – cranial and caudal – and the rods are mounted with pedicle screws. For cases involving the pelvis (mostly neuromuscular), ilio-sacral screws (Tanit, Euros, La Ciotat, France) are used. For hybrid constructs (Fig. 2a), an additional small incision is made for the apical screw(s). The rod is carefully contoured in both the coronal and sagittal plane. Then, the side-to-side connectors with an oversized hole for sliding are temporarily fixed to the sliding rod, leaving a residual rod length of 4–6 cm. The spring is pre-tensioned against the side-to-side connectors with a buttress. The sliding rod is then inserted sub-fascially and mounted to the anchors. Prophylactic intra-wound vancomycin powder is used routinely [24]. An example of a surgery in an idiopathic EOS patient can be seen in Video 1. Postoperatively, patients are not restricted in mobilization and do not need to wear a brace.

Since the first development of the SDS, several improvements have been implemented in its design. After investigating common SDS complications, we changed to an SDS configuration with 5.5 mm rods instead of 4.5 mm rods and started using stacked side-to-side connectors to minimize implant kyphosis, which reduced the complication rate compared to the earlier version [21, 25]. In addition, we started using longer springs to limit the need for retensioning, and differential spring forces (i.e., stronger springs on the curve concavity vs. the curve convexity). This changes were implemented around the end of 2019, which means that the current study includes patients before and after these changes.

### Outcome parameters

Baseline demographics and surgical characteristics (skin-to-skin surgery time, estimated blood loss and days until discharge) were prospectively recorded. Using the complete C-EOS classification (including Major curve angle, kyphosis and progression rate), resulted in too many subgroups to make meaningful comparisons. Therefore, we only subdivided patients based on the main four etiological groups. Radiographic measurements by three investigators (CST, IEB and JVCL) included coronal Cobb angles, T5-T12 kyphosis, L1-S1 lordosis and the T1-T12 and T1-S1 heights. Radiographs were not blinded for follow-up time. Heights were determined with Surgimap v.2.3.2.1 (Nemaris Inc, NY, US) by drawing a perpendicular line between two horizontal lines through the midpoints of the vertebral endplates. Coronal and sagittal heights were averaged. Length gain was calculated as the difference between preoperative and postoperative length, as well as ‘true’ growth, from the initial postoperative erect radiograph until the latest follow-up, excluding the height gain from the initial and final fusion surgery. All radiographs were calibrated using rod diameters (4.5 mm or 5.5 mm).

### Complications and unplanned return to the operating room (UPROR)

Complications and UPRORs were recorded prospectively according to protocol and classified using the Clavien-Dindo-Sink (CDS) grading system, which was shown to be reproducible in AIS and EOS populations [26, 27]. Prospectively, only CDS Grades 2 and above were collected. However, we retrospectively reviewed the patient files and imaging data to find implant-related complications that were not prospectively scored. We distinguished complications that were disease-related (e.g., respiratory insufficiency in neuromuscular patients), surgery-related (e.g., deep surgical site infection) or implant-related (e.g., rod fracture). Implant re-tensioning due to excessive spinal growth was not considered a complication, as growth is desirable, but was recorded as an UPROR. Any additional surgery related to SDS treatment until final fusion or end of growth was considered an UPROR. Final fusion after the arbitrary age of 12 years was not deemed an UPROR as this is anticipated. All implant-related complications that were or will be treated during final fusion were scored as CDS 3. A flowchart of how we scored complications and UPRORs can be found in Appendix 1.

### Statistical analysis

Normally distributed data are presented as mean and standard deviation. A one-way ANOVA was used to compare baseline characteristics and complications and UPRORs across etiologies. Since there were only few syndromic patients, this group was excluded from the etiology comparison. Post-hoc testing with correction for multiple testing was performed using the Holm-Bonferroni procedure. Changes over time were compared in two steps. First, the difference between preoperative and directly postoperative was assessed and compared using a mixed repeated-measures ANOVA. Second, changes from postoperative measurements until latest follow-up (before EOS graduation) were analyzed using linear mixed models (LMMs). This method allows for integration of data from patients that had missing data points or different follow-up. To determine specific effects, a restricted maximum likelihood estimator model was created for each outcome with gender, preoperative value, follow-up time, and etiology as fixed effects. Patient ID was added as a random effect. In addition, the interaction between follow-up time and etiology was added as a fixed effect to identify whether changes over time differed between etiologies. For each model, we evaluated whether the model had increased predictive performance when using a non-linear association compared to a linear association, using the Akaike Information Criterion. If no differences were found, a standard linear relationship model was used.

Event-free survival of complications and UPRORs was visualized using a Kaplan–Meier curve, and compared between groups using a Cox proportional hazard model, if the required assumptions were met. Statistical analysis was performed in SPSS version 29.0.1.0 (IBM SPSS Statistics, Armonk, NY, USA). The LMMs were conducted in R Statistical software version 4.0.2 (R Foundation for Statistical Computing, Vienna, Austria). Data visualization was performed using GraphPad Prism version 10.1.2 (GraphPad Software, LLC, Boston, MA, USA). Statistical significance was defined as *p* < 0.05.

## Results

### Patient demographics

A total of 68 patients were reviewed: 14 congenital/structural (hereafter referred to as “congenital”), 41 neuromuscular, 4 syndromic, and 9 idiopathic. Due to the small number of syndromic patients, this group was excluded from further analysis. An overview of all etiologies and underlying pathologies is shown in Appendix 2. A total of 25 patients graduated from SDS treatment after a mean follow-up of 3.2 years, with 23 patients undergoing a final fusion, and two patients where the SDS was left in situ. Baseline characteristics as well as the SDS strategy are summarized in Table 1. Mean age at surgery was 8.4 ± 1.7 years. Mean follow-up was 3.7 ± 1.4 years. The preoperative major curve was 73 ± 18°, with no significant differences between groups. Similarly, T5-T12 kyphosis was 25° and L1-S1 lordosis was 49°. Surgical duration, estimated blood loss and time to discharge were also similar across all etiologies. As expected, neuromuscular patients had significantly longer constructs (16 levels) compared to congenital (13 levels) or idiopathic (12 levels) patients. There were four unilateral constructs (all congenital patients), 25 hybrid constructs (in 9 congenital, 7 neuromuscular, and 9 idiopathic patients), and 35 bilateral constructs (in one congenital and 34 neuromuscular patients). A more detailed overview of instrumentation strategies is shown in Table 1 and Appendix 2.

**Table 1:** Baseline characteristics

	All (*N* = 64)	Congenital (*N* = 14)	Neuromuscular (*N* = 41)	Idiopathic (*N* = 9)	*p* value
Age at surgery (years)	8.4 ± 1.7	7.6 ± 2.0	8.4 ± 1.5	9.7 ± 1.7^b^	0.017
Girls (%)	29 (45%)	8 (57%)^c^	14 (34%)	7 (78%)^c^	0.035
Preoperative primary curve (°)	73 ± 18	75 ± 26	73 ± 13	67 ± 16	0.566
Preoperative secondary curve (°)	37 ± 16	45 ± 15	33 ± 16	42 ± 8	0.156
Preoperative T5-T12 kyphosis (°)	25 ± 20	28 ± 29	24 ± 23	29 ± 14	0.761
Preoperative L1-S1 lordosis (°)	49 ± 19	55 ± 15	46 ± 21	53 ± 12	0.257
Preoperative T1-T12 height (mm)	168 ± 29	142 ± 23	173 ± 23^b^	191 ± 21^b^	< 0.001
Preoperative T1-S1 height (mm)	284 ± 41	264 ± 33	282 ± 40	325 ± 31^b,c^	0.001
Surgical duration (min)	189 ± 45	187 ± 58	190 ± 44	182 ± 30	0.880
Estimated blood loss (mL)	385 ± 191	409 ± 203*	382 ± 181	367 ± 238	0.880
Instrumented levels	15 ± 3	13 ± 4	16 ± 2^b,d^	12 ± 2	< 0.001
Instrumented to pelvis	33 (52%)	0	33 (80%)^b,d^	0	< 0.001
Free vertebrae within construct	10 ± 3	7 ± 3	12 ± 2^b,d^	7 ± 2	< 0.001
Time to discharge (days)	7 ± 6	7 ± 4	7 ± 7	5 ± 1	0.654
Follow-up length (years)	3.7 ± 1.4	4.5 ± 1.6^c,d^	3.5 ± 1.4	3.3 ± 0.7	0.012
Implant configuration					< 0.001
Unilateral construct	4	4	0	0	
Concave 75 N	3	3	0	0	
Concave 100 N	1	1	0	0	
Bilateral construct	35	1	34	0	
Concave 75 N, Convex 75 N	16	1	15^a^	0	
Concave 100 N, Convex 50 N	19	0	19	0	
Hybrid construct	25	9	7	9	
Concave 75 N, Convex 0 N	17	7	4	6	
Concave 100 N, Convex 0 N	8	2	3	3	

Post-hoc testing with correction for multiple testing through the Holm-Bonferroni procedure

^*^Estimated blood loss data was missing in three congenital patients

^a^In two neuromuscular patients the springs were partially compressed perioperatively (60 N remaining)

^b^Significantly greater compared to the congenital group

^c^Significantly greater compared to the neuromuscular group

^d^Significantly greater compared to the idiopathic group

### Curve outcomes

**Fig. 3:**
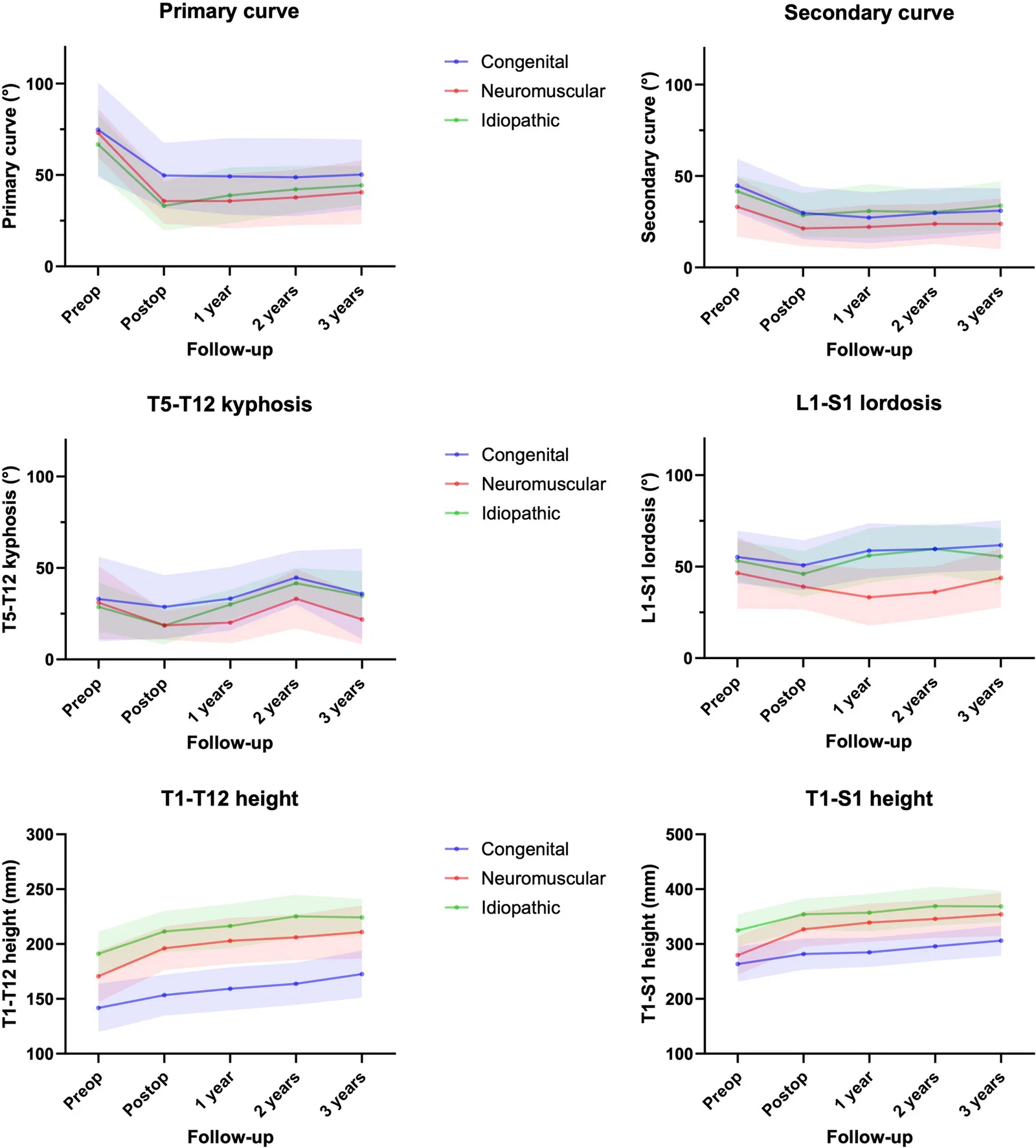
Coronal curves, sagittal curves, T1-T12 and T1-S1 height over time: Measurements (mean ± SD) at group level up to three years of follow-up

**Fig. 4:**
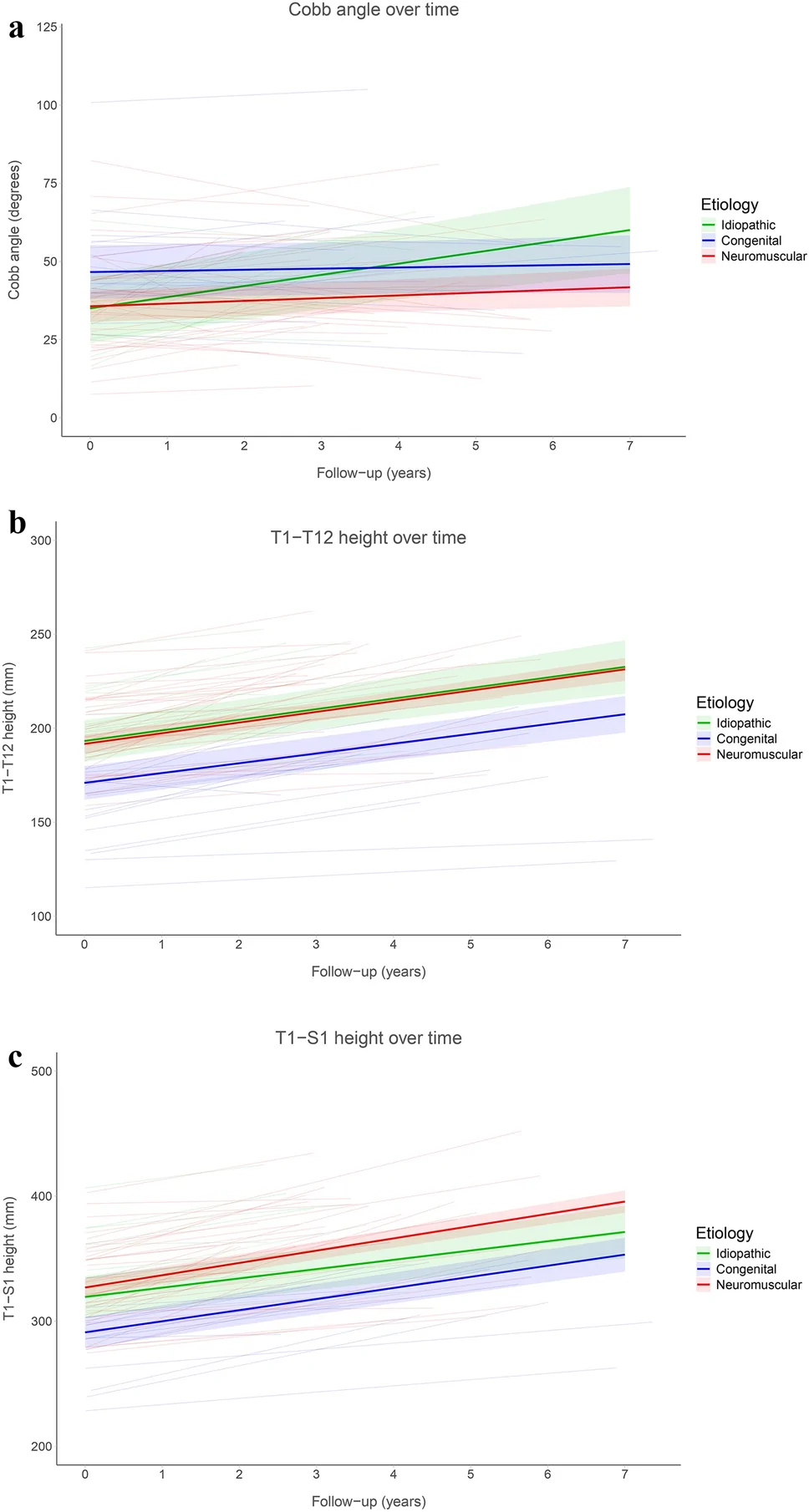
a. Linear mixed model results of main coronal Cobb angle over time. b. Linear mixed model results of T1-T12 height over time. c. Linear mixed model results of T1-S1 height over time

Curve changes over time for each group are shown in Fig. 3. The overall postoperative main curve correction was 47% (Table 2). For congenital patients, this correction was 33%, while neuromuscular and idiopathic patients achieved an average correction of 51%. The LMM investigating the interaction between etiology and follow-up (Fig. 4a, Table 3) showed that idiopathic EOS patients had an increase in Cobb angle after the correction of 3.6°/year, whereas congenital and neuromuscular patients showed a significantly lower increase, 0.4°/year and 0.9°/year, respectively.

**Table 2:** Curve characteristics and spinal height per etiology over time

	Etiology	Preoperative	Postoperative	Change (%)	*p* value between groups
Primary curve (°)	Congenital	75 ± 27	50 ± 19	− 25 (− 33%)	0.023
	Neuromuscular^a^	73 ± 15	36 ± 13	− 37 (− 51%)	
	Idiopathic^a^	67 ± 15	33 ± 14	− 34 (− 51%)	
Secondary curve (°)	Congenital	41 ± 19	28 ± 16	− 13 (− 32%)	0.896
	Neuromuscular	33 ± 17	21 ± 10	− 12 (− 36%)	
	Idiopathic	42 ± 8	28 ± 13	− 14 (− 33%)	
T5-T12 kyphosis (°)	Congenital^b^	28 ± 30	33 ± 18	+ 5 (+ 18%)	0.042
	Neuromuscular	24 ± 23	16 ± 14	− 8 (− 33%)	
	Idiopathic	29 ± 14	30 ± 9	+ 1 (+ 3%)	
L1-S1 lordosis (°)	Congenital	55 ± 15	51 ± 14	− 4 (− 7%)	0.938
	Neuromuscular	46 ± 21	40 ± 13	− 6 (− 13%)	
	Idiopathic	53 ± 12	46 ± 13	− 7 (− 13%)	
T1-T12 height (mm)	Congenital	142 ± 23	153 ± 19	+ 11 (+ 8%)	0.055
	Neuromuscular	173 ± 24	196 ± 20	+ 23 (+ 13%)	
	Idiopathic	191 ± 21	211 ± 20	+ 20 (+ 10%)	
T1-S1 height (mm)	Congenital	264 ± 33	282 ± 29	+ 18 (+ 7%)	< 0.001
	Neuromuscular^a,c^	280 ± 36	327 ± 32	+ 45 (+ 16%)	
	Idiopathic^a^	325 ± 31	354 ± 30	+ 29 (+ 9%)	

Post-hoc testing with correction for multiple testing through the Holm-Bonferroni procedure

^a^Change is significantly greater compared to the congenital group

^b^Change is significantly greater compared to the neuromuscular group

^c^Change is significantly greater compared to the idiopathic group

**Table 3:** Linear mixed model results of main coronal Cobb angle over time

Predictors	Estimates	95% CI	*p* value
Female	1.9	− 5.5 to 9.3	0.607
Preoperative Cobb angle (°)	0.4	0.2 to 0.6	< 0.001
Postoperative Cobb angle (°)^a^	5.5	− 11.1 to 22.0	0.516
Postoperative Cobb angle across etiology (°)^a^			
Idiopathic	Reference	Reference	Reference
Congenital	11.6	− 1.0 to 24.2	0.070
Neuromuscular	0.7	− 10.6 to 11.9	0.908
Cobb angle change per year (°)^a^	3.6	1.9 to 5.2	< 0.001
Cobb angle change per year across etiology (°)^a^			
Idiopathic	Reference	Reference	Reference
Congenital	− 3.2	− 5.1 to − 1.4	< 0.001
Neuromuscular	− 2.7	− 4.5 to − 0.9	0.003

The graph shows the mean line per etiology (based on a male with the median preoperative Cobb angle) and the individual patient lines. For clarity, the individual lines are shown as straight lines between only the first and last postoperative follow-up. For the statistical analysis, all follow-up points in between were also considered. Using the above predictors (gender, preoperative Cobb angle and etiology), the follow-up Cobb angle can be predicted for patients undergoing SDS treatment. The marginal R2 value of the above model was 0.67, outlining that the above predictors could predict 67% of the variation between data points

Example: What is the expected main coronal Cobb angle after 3 years for a female congenital EOS patient with a preoperative main coronal Cobb angle of 70°? a) Female: Add 1.9° if female. b) Preoperative Cobb angle: Start with 0.4° for every preoperative 1.0°.c) Postoperative Cobb angle: Add 5.5°. d) Postoperative Cobb angle across etiology: Add 11.6° if congenital. e) Cobb angle change per year: Add 3.6° for every year. f) Cobb angle change per year across etiology: Subtract 3.2° for every year if congenital. The total expected main coronal Cobb angle becomes: 1.9 + 28 + 5.5 + 11.6 + 10.8 – 9.6 = 48.2°

^a^The initial change is added for all groups, but for the congenital and neuromuscular group, additional changes are later added. Since the idiopathic group is the reference group, and no changes are added for the idiopathic group, the initial changes are identical to changes in the idiopathic group

Immediately following surgery, T5-T12 kyphosis was reduced in neuromuscular patients (−8°, −33%), increased somewhat in congenital patients (+ 5°, + 18%), while kyphosis remained stable in idiopathic patients (+ 1°, + 3%). L1-S1 lordosis was mildly reduced postoperatively in all groups, with no differences between groups. During follow-up, kyphosis remained stable in congenital and neuromuscular patients (Appendix 3). In idiopathic patients, kyphosis increased during the first years, especially when compared to neuromuscular patients (*p* = 0.03). For L1-S1 lordosis, all patients showed a tendency of increasing lordosis, with no differences between groups (Appendix 4).

### Spinal length gain

Spinal length data are presented in Table 2, Table 4, Table 5, and Fig. 4b/c. Immediately following SDS implantation, increases in T1-T12 height were observed (due to curve reduction and soft-tissue lengthening), which were largest in neuromuscular patients (+ 23 mm, 13%), followed by idiopathic patients (+ 20 mm, 10%) and congenital patients (+ 11 mm, 8%). For T1-S1, the increase in the neuromuscular group (+ 45 mm, 16%) was significantly greater than in both the idiopathic group (+ 29 mm, 9%) and the congenital group (+ 18 mm, 7%).

**Table 4:** Linear mixed model results of T1-T12 height over time

Predictors	Estimates	95% CI	*p* value
Female	0.7	−6.3 to 7.7	0.839
Preoperative T1-T12 height (mm)	0.7	0.6 to 0.8	< 0.001
Postoperative T1-T12 height (mm)^a^	79.4	51.3 to 107.4	< 0.001
Postoperative T1-T12 height across etiology (mm)^a^			
Idiopathic	Reference	Reference	Reference
Congenital	− 22.3	− 36.1 to -8.6	0.002
Neuromuscular	− 1.6	− 12.5 to 9.4	0.779
T1-T12 height change per year (mm)^a^	5.6	3.8 to 7.5	< 0.001
T1-T12 height change per year across etiology (mm)^a^			
Idiopathic	Reference	Reference	Reference
Congenital	− 0.4	− 2.5 to 1.6	0.689
Neuromuscular	0.0	− 1.9 to 2.0	0.969

The graph shows the mean line per etiology (based on a male with the median preoperative T1-T12 height) and the individual patient lines. For clarity, the individual lines are shown as straight lines between only the first and last postoperative follow-up. For the statistical analysis, all follow-up points in between were also considered. Using the above predictors (gender, preoperative T1-T12 height and etiology), the follow-up T1-T12 height can be predicted for patients undergoing SDS treatment. The marginal R2 value of the above model was 0.93, outlining that the above predictors could predict 93% of the variation between data points

Example: What is the expected T1-T12 height after 5 years for a male neuromuscular EOS patient with a preoperative T1-T12 height of 180 mm?. a) Male: Add nothing if male. b) Preoperative T1-T12 height: Start with 0.7 mm for every preoperative 1.0 mm. c) Postoperative T1-T12 height: Add 79.4 mm. d) Postoperative T1-T12 height across etiology: Subtract 1.6 mm if neuromuscular. e) T1-T12 height change per year: Add 5.6 mm for every year. f) T1-T12 height change per year across etiology: Add 0 mm for every year if neuromuscular. The total expected T1-T12 height becomes: 126 + 79.4 - 1.6 + 28 + 0 = 231.8 mm

^a^The initial change is added for all groups, but for the congenital and neuromuscular group, additional changes are later added. Since the idiopathic group is the reference group, and no changes are added for the idiopathic group, the initial changes are identical to changes in the idiopathic group

**Table 5:** Linear mixed model results of T1-S1 height over time

Predictors	Estimates	95% CI	*p* value
Female	1.4	− 9.0 to 11.9	0.785
Preoperative T1-S1 height (mm)	0.7	0.6 to 0.9	< 0.001
Postoperative T1-S1 height (mm)^a^	115.5	69.4 to 161.5	< 0.001
Postoperative T1-S1 height across etiology (mm)^a^			
Idiopathic	Reference	Reference	Reference
Congenital	− 28.3	− 48.0 to -8.7	0.005
Neuromuscular	7.6	− 9.4 to 24.5	0.376
T1-S1 height change per year (mm)^a^	7.4	4.8 to 10.0	< 0.001
T1-S1 height change per year across etiology (mm)^a^			
Idiopathic	Reference	Reference	Reference
Congenital	1.5	− 1.4 to 4.4	0.321
Neuromuscular	2.4	− 0.4 to 5.2	0.093

The graph shows the mean line per etiology (based on a male with the median preoperative T1-S1 height) and the individual patient lines. For clarity, the individual lines are shown as straight lines between only the first and last postoperative follow-up. For the statistical analysis, all follow-up points in between were also considered. Using the above predictors (gender, preoperative T1-S1 height and etiology), the follow-up T1-S1 height can be predicted for patients undergoing SDS treatment. The marginal R2 value of the above model was 0.93, outlining that the above predictors could predict 93% of the variation between data points

^a^The initial change is added for all groups, but for the congenital and neuromuscular group, additional changes are later added. Since the idiopathic group is the reference group, and no changes are added for the idiopathic group, the initial changes are identical to changes in the idiopathic group

The amount of “true growth” during follow-up, for both T1-T12- and T1-S1 segment were excellently predicted using LMMs, after correcting for gender, etiology and preoperative height with R^2^ values of 0.93. T1-T12 height increased at an average rate of 5.6 mm/year, with no significant differences between etiologies. In the LMM, T1-S1 growth was 7.4 mm/year in the idiopathic patients. In congenital and neuromuscular patients, growth was slightly larger although the differences were not statistically significant (8.9 mm/year and 9.8 mm/year, respectively). As the trends over time were best described by linear rather than exponential relationships, no evidence of time-dependent diminishing growth was found across the entire follow-up period in any of the groups.

### Complications and unplanned returns to the operating room (UPRORs)

A comprehensive overview of complications CDS ≥ 2 and UPRORs is provided in Appendix 5. A total of 59 complications occurred corresponding to 0.25 complications/patient/year. The mean time to complications was 1.9 years. The congenital group experienced 14 complications (0.22/patient/year), the neuromuscular group 37 (0.26/patient/year), and the idiopathic group 8 (0.27/patient/year). Within the first two years of follow-up, a first complication occurred in 36% of congenital patients, 32% of neuromuscular patients and 33% of idiopathic patients.

The most frequent were implant-related (*N* = 42), primarily rod fractures of the initially used 4.5 mm rods and excessive kyphosis of the system. Surgery-related complications (*N* = 10) were heterogeneous, most commonly deep surgical site infection (*N* = 4). Disease-related complications (*N* = 7) included four patients with 5 episodes of pulmonary insufficiency requiring hospitalization. In addition, two neuromuscular patients died of respiratory failure unrelated to the implant and long after the initial surgery.

**Fig. 5:**
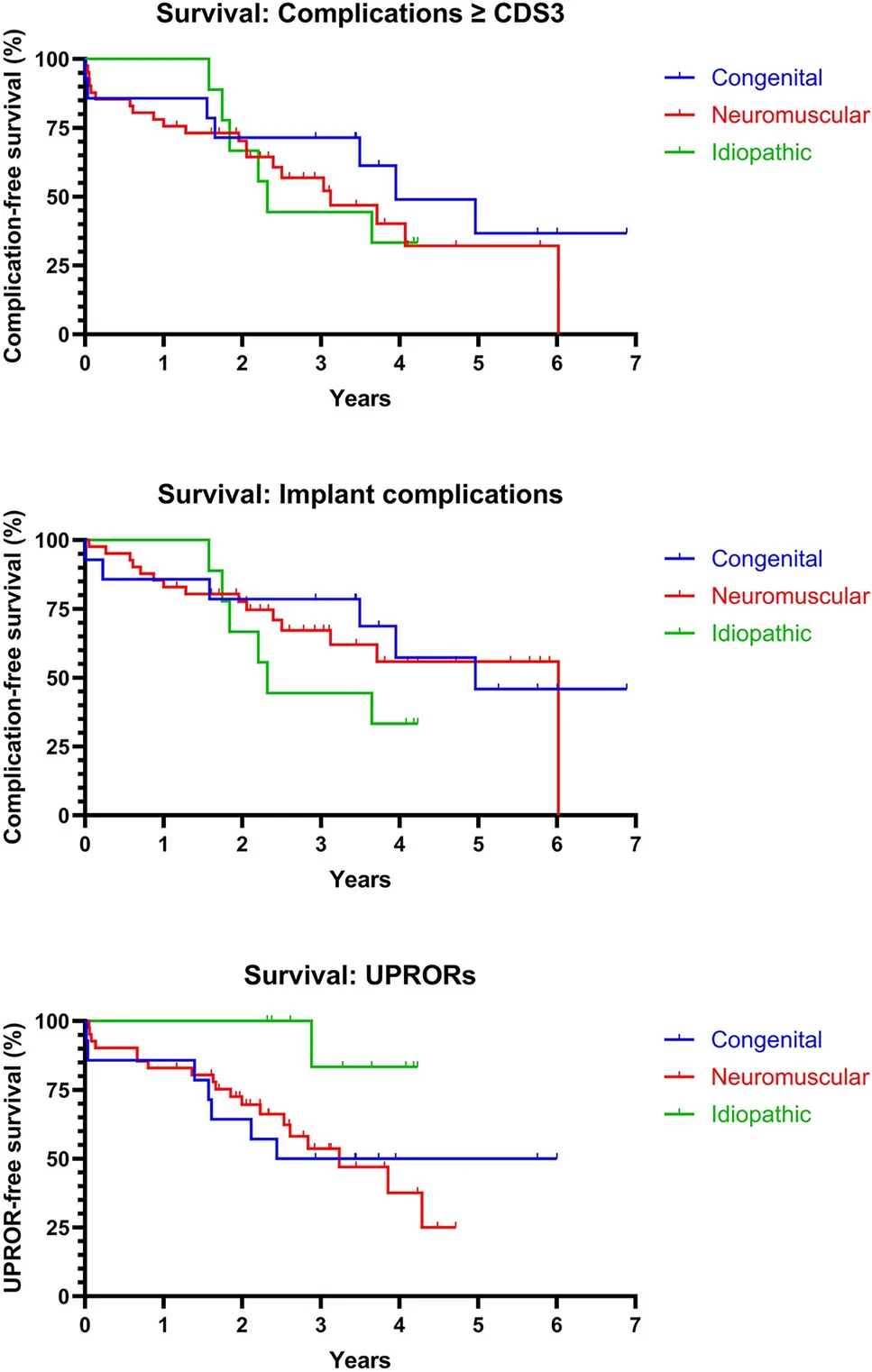
Survival analysis of complications and UPRORs. Survival time to the occurrence of **a** Complications with severity CDS 3 and higher, **b** Implant-related complications, **c** UPRORs. Ticks denote censored patients

A total of 42 UPRORs were recorded, corresponding to 0.18 UPROR/patient/year (Table 6). Congenital and idiopathic patients had the lowest rates, with 0.19 and 0.07 UPROR/patient/year, respectively. Neuromuscular patients experienced the highest rate of 0.20 UPROR/patient/year. The most common UPROR was spring re-tensioning surgery (*N* = 10), which was performed in 3 congenital and 7 neuromuscular patients after an average interval of 1.9 years post-index surgery. In total, 15 posterior spinal fusions were performed. In four cases, the fusion was performed < 12 years of age and deemed an UPROR. With the complication and UPROR data, survival graphs were created for occurrence of complications ≥ CDS 3 (Fig. 5a), occurrence of implant complications (Fig. 5b) and UPRORs (Fig. 5c). Statistical comparison between groups using a Cox proportional hazard model was not performed, as the proportional hazard assumption was not met.

## Discussion

This study broadens the understanding of the efficacy of SDS treatment across various EOS etiologies. Although different strategies were used, we consider the SDS (irrespective of the configuration) as a concept and attempted to compare performance of this concept for the different etiologies. In general, the results align with previous reports demonstrating the efficacy of the technique and relatively low complication rates [20, 21, 25]. Notably, neuromuscular patients, a vulnerable subgroup, appeared to benefit most from SDS treatment. The ability to control the curve in idiopathic EOS patients on the other hand is not yet optimal. It could be hypothesized that the underlying mechanism, along with increased activity levels and muscle tone in idiopathic patients, are more powerful and less susceptible to the corrective distraction forces that were used. These forces were probably too low for these patients, due to the hybrid configuration with only one 75N spring, instead of a bilateral SDS that typically involves a total of 150N of distraction force. Based on our systematic review and meta-analysis, the use of stronger springs in older (idiopathic) patients is safe, which is why we changed the treatment strategy and currently use 100N or even 150N springs [28]. Future research will need to test whether this configuration change improves correction over time in larger patient groups.

A recent study by Grabala et al. presented the long-term outcomes of an international cohort of patients treated with the MCGR, performing similar subgroup analyses for different etiologies. They reported an improvement in the main coronal curves across all subgroups of approximately 47%, however, this included the results after final fusion. Their analysis of spinal height increase also included the patients who had undergone final fusion, which affects results and complicates comparisons between techniques or etiologies [29]. Fortunately, the authors provided relevant data suitable for comparison with this current study [30]. They observed true (i.e., after insertion and before final fusion) T1-T12 growth of 6.2 mm/year in idiopathic patients, 5.5 mm/year in congenital patients, and 5.2 mm/year in neuromuscular patients, which is comparable to our observations. Literature on T1-T12 and T1-S1 growth rates in patients treated with TGR or MCGR varies widely, ranging from 2–23 mm/year [31]. Due to the heterogeneity of datapoints in this study, spinal growth was best described using LMMs. Interestingly, this method revealed that T1-T12 and T1-S1 growth did not diminish over time, suggesting that the SDS does not suffer from the “law of diminishing returns”, at least when regarding the first four years follow-up [18].

We used the CDS classification system, which is easy to use and reproducible. A major disadvantage is that this score does not take into account that the final fusion surgery is part of the “growth-friendly” strategy, and can thus be used as an opportunity to address implant-related complications that arise during “growth-friendly” treatment. For example, if an older EOS patient with SDS experiences a unilateral rod fracture, with no clinical complaints, a sensible strategy would be to wait until final fusion surgery, where this complication can then be addressed. However, whether treating this complication then counts as an UPROR, or even as a CDS grade 3, can be argued. For completeness, in this study, all implant-related complications, that were, or will be addressed at final fusion surgery were graded as CDS 3. The most frequent complications were implant-related (72%), mainly due to rod fractures (23%), highlighting room for improvement. Indeed, transitioning to 5.5 mm rods reduced these drastically, with only one rod fracture observed in 19 patients with an average follow-up of 2.5 years. Spring re-tensioning was the most common reason for UPRORs (23%). This was a reason to change to longer springs when possible which, due to the lower spring constant, maintain distraction force for a longer period. Future studies will have to confirm this effect. Re-tensioning was not classified as a complication in this study, as it is a consequence of extensive growth, which is considered favourable. This extra length gain – of up to 1.6 cm – above physiological growth results partly from the initial viscoelastic effect (i.e., creep) after insertion and before the first erect radiograph, this was not included in measured height gain.

**Table 6:** Overview of complications and UPRORs per etiology

	Congenital	Neuromuscular	Idiopathic	Total
Complications	14	37	8	59
CDS 2	2	1	0	3
CDS 3	12	31	8	51
CDS 4	0	3	0	3
CDS 5	0	2	0	2
Complications/patient	1.0	0.90	0.89	0.92
Complications/patient/year	0.22	0.26	0.27	0.25
UPRORs	12	28	2	42
UPROR/patient	0.86	0.68	0.22	0.66
UPROR/patient/year	0.19	0.20	0.07	0.18

The overall UPROR rate was 0.18/patient/year, lowest in idiopathic patients (0.07/patient/year), and highest in neuromuscular patients (0.20/patient/year). A recent study by McIntosh et al. reported a similar UPROR rate of 0.20/patient/year in patients treated with the MCGR [32]. This higher UPROR rate in neuromuscular patients was expected based on literature and is related to a more vulnerable patient population with a higher risk of infection and a higher susceptibility to implant prominence due to kyphosis as they generally have less soft tissue coverage. This tendency for kyphosis has been addressed using two stacked parallel connectors instead of one and was shown to be effective in a previous study [21]. It should be mentioned that the definition of UPROR (and complication) influences these numbers; definitive spinal fusion at age ≥ 12 years for example was arbitrarily not considered an UPROR, if we set this threshold at 13 years there would be four additional final fusions included as UPRORs, increasing the rate to 0.21/patient/year. During several UPRORs, considerable metallosis around the sliding anchors was observed. Currently, implant improvements are being developed that aim to minimize metallosis in the future.

Although, this current study was not designed for comparison to other “growth-friendly” systems, regarding curve correction, spinal growth, complications and UPROR rates, the performance of the SDS seems comparable and satisfactory. An appealing opportunity with the SDS is that this technique is just the beginning of dynamic growth guidance, offering tremendous room for improvement. For example, many strategic configurations may be used, and identifying optimal spring forces may improve outcomes. Furthermore, the SDS design supports personalized treatment strategies, allowing adjustments in spring length and force, tailored to specific EOS etiologies and curve types.

A limitation of this study is the small number of patients per etiology. Therefore, the conclusions based on these relatively small cohorts should be interpreted with caution. In addition, many implant configurations were (almost) exclusively used in specific etiological groups. Therefore, the differences found between groups could have been caused by either (a combination of) curve etiology, or by the employed implant configuration. We also implemented several changes to reduce the number of complications during the study, this leads to treatment heterogeneity and precludes identification of one optimal strategy. Nevertheless, we regard the SDS as an evolving concept that is constantly improved while used for different etiologies of EOS. Unfortunately, syndromic scoliosis was hardly represented, which is due to the safety policy of excluding patients with compromised tissue integrity and bone strength. Moreover, the case mix in this study may not fully represent the broader population of EOS patients, as our academic hospital is a specialized center for specific neuromuscular patients, like spinal muscular atrophy. As these patients are hypotonic, results are not easily generalized to more spastic neuromuscular patients. Finally, although this is a multicenter study, most patients (> 90%) come from the academic hospital where the technique was invented.

## Conclusion

The current study shows that for different EOS etiologies, the SDS performs comparable to other “growth-friendly” systems in terms of curve correction, spinal growth and complications, but without additional lengthening procedures. Initial curve correction is highest in neuromuscular and idiopathic patients, and lowest in congenital patients. Spinal growth during the first four years was similar for all etiologies. During follow-up, the SDS appeared less capable to maintain correction for idiopathic patients. Complication rate was similar between etiologies, idiopathic patients had the lowest UPROR rate.

## Electronic supplementary material

Below is the link to the electronic supplementary material.

Supplementary file1 (EPS)—Complication and UPROR flowchart

Supplementary file2 (DOCX 22 KB) - Underlying pathology and implant configuration

Supplementary file3 (DOCX 329 KB) - Linear mixed model results of T5-T12 kyphosis over time

Supplementary file4 (DOCX 343 KB) - Appendix 4. Linear mixed model results of L1-S1 lordosis over time

Supplementary file5 (DOCX 41 KB) - Patient complications and UPROR data

Supplementary file6 Video. Spring Distraction System surgeryz (MP4 49238 KB)

## Data Availability

Data is available upon reasonable request.
