# Supplementary material for: Efficacy of the spring distraction system for different etiologies of early onset scoliosis: evaluating an evolving treatment concept
Source: Spine Deform. 2026 Jun 2;14(5):1921–33. doi: 10.1007/s43390-026-01368-0 (PMC13553710; doi:10.1007/s43390-026-01368-0)
Supplement: Supplementary file 1 [file 43390_2026_1368_MOESM2_ESM.docx]

| **Appendix 2. Underlying pathology and implant configuration** | | | | | |
| --- | --- | --- | --- | --- | --- |
| **Patient** | **Etiology** | **Underlying pathology** | **SDS configuration** | **Spring force** | |
|  |  |  |  | **Concave** | **Convex** |
| **P1** | C | Unilateral hemivertebra | Unilateral | 75 N |  |
| **P2** | C | Spondylocarpotarsal synostosis syndrome | Bilateral | 75 N | 75 N |
| **P3** | C | Klippel-Feil syndrome | Hybrid | 75 N |  |
| **P4** | I |  | Hybrid | 75 N |  |
| **P5** | NM | Spina bifida | Hybrid | 75 N |  |
| **P6** | C | Thoracolumbar hemivertebrae | Unilateral | 75 N |  |
| **P7** | NM | Spina bifida | Bilateral | 75 N | 75 N |
| **P8** | I |  | Hybrid | 75 N |  |
| **P9** | NM | Ullrich congenital muscular dystrophy | Hybrid | 75 N |  |
| **P10** | NM | Spina bifida | Bilateral | 75 N | 75 N |
| **P11** | NM | Congenital myopathy | Bilateral | 75 N | 75 N |
| **P12** | NM | SMA type II | Bilateral | 60 N | 60 N |
| **P13** | NM | Congenital myopathy | Bilateral | 75 N | 75 N |
| **P14** | C | Klippel-Feil syndrome | Hybrid | 75 N |  |
| **P15** | C | Hemivertebrae, block vertebrae | Hybrid | 75 N |  |
| **P16** | NM | Myasthenia Gravis | Bilateral | 75 N | 60 N |
| **P17** | NM | SMA type II | Bilateral | 75 N | 75 N |
| **P18** | I |  | Hybrid | 75 N |  |
| **P19** | NM | Brain-lung-thyroid syndrome | Hybrid | 75 N |  |
| **P20** | NM | Bohring-Opitz syndrome | Bilateral | 75 N | 75 N |
| **P21** | C | Hemivertebrae | Unilateral | 75 N |  |
| **P22** | NM | SMA type II | Bilateral | 75 N | 75 N |
| **P23** | NM | Cerebral palsy | Bilateral | 75 N | 75 N |
| **P24** | NM | SMA type II | Bilateral | 75 N | 75 N |
| **P25** | NM | SMA type II | Bilateral | 75 N | 75 N |
| **P26** | NM | Spina bifida | Bilateral | 75 N | 75 N |
| **P27** | NM | Chondrodysplasia punctata | Hybrid | 75 N |  |
| **P28** | I |  | Hybrid | 75 N |  |
| **P29** | C | VACTERL | Hybrid | 75 N |  |
| **P30** | I |  | Hybrid | 75 N |  |
| **P31** | C | Hemivertebrae | Hybrid | 75 N |  |
| **P32** | NM | SMA type II | Bilateral | 75 N | 75 N |
| **P33** | NM | Emery-Dreifuss muscular dystrophy | Bilateral | 75 N | 75 N |
| **P34** | NM | SMA type II | Bilateral | 100 N | 50 N |
| **P35** | I |  | Hybrid | 100 N |  |
| **P36** | I |  | Hybrid | 75 N |  |
| **P37** | NM | 15q syndrome | Hybrid | 100 N |  |
| **P38** | I |  | Hybrid | 100 N |  |
| **P39** | NM | SMA type II | Bilateral | 100 N | 50 N |
| **P40** | NM | SMA type II | Bilateral | 100 N | 50 N |
| **P41** | C | VACTERL | Hybrid | 75 N |  |
| **P42** | I |  | Hybrid | 100 N |  |
| **P43** | NM | Spinal cord glioma | Bilateral | 100 N | 50 N |
| **P44** | C | Arthrogryposis multiplex congenita | Hybrid | 100 N |  |
| **P45** | C | Chromosomal anomaly | Hybrid | 75 N |  |
| **P46** | NM | Williams syndrome | Hybrid | 100 N |  |
| **P47** | C | Spondylocostal dysostosis | Unilateral | 100 N |  |
| **P48** | NM | SMA type II | Bilateral | 100 N | 50 N |
| **P49** | C | Arthrogryposis multiplex congenita | Hybrid | 100 N |  |
| **P50** | NM | Spina bifida | Hybrid | 100 N |  |
| **P51** | NM | Spina bifida | Bilateral | 100 N | 50 N |
| **P52** | NM | SMA type II | Bilateral | 100 N | 50 N |
| **P53** | NM | SMA type Ic | Bilateral | 100 N | 50 N |
| **P54** | NM | 22q11 deletion syndrome | Bilateral | 100 N | 50 N |
| **P55** | NM | Chomosomal translocation | Bilateral | 100 N | 50 N |
| **P56** | NM | SMA type II | Bilateral | 100 N | 50 N |
| **P57** | NM | SMA type II | Bilateral | 100 N | 50 N |
| **P58** | NM | SMA type II | Bilateral | 100 N | 50 N |
| **P59** | NM | SMA type Ic | Bilateral | 100 N | 50 N |
| **P60** | NM | West syndrome | Bilateral | 100 N | 50 N |
| **P61** | NM | SMA type II | Bilateral | 100 N | 50 N |
| **P62** | NM | Cerebral palsy | Bilateral | 100 N | 50 N |
| **P63** | NM | Congenital myopathy | Bilateral | 100 N | 50 N |
| **P64** | NM | SMA type II | Bilateral | 100 N | 50 N |
| C: congenital; NM: neuromuscular; I: idiopathic; SMA: spinal muscular atrophy; VACTERL vertebral defects, anal atresia, cardiac defects, trachea-esophageal fistula, renal anomalies, limb abnormalities | | | | | |
