# Supplementary material for: Efficacy of the spring distraction system for different etiologies of early onset scoliosis: evaluating an evolving treatment concept
Source: Spine Deform. 2026 Jun 2;14(5):1921–33. doi: 10.1007/s43390-026-01368-0 (PMC13553710; doi:10.1007/s43390-026-01368-0)
Supplement: Supplementary file 2 [file 43390_2026_1368_MOESM3_ESM.docx]

| **Appendix 3. Linear mixed model results of T5-T12 kyphosis over time** | | | | |
| --- | --- | --- | --- | --- |
| **Predictors** | | **Estimates** | **95% CI** | ***p* value** |
| Female | | -2.4 | -6.3 to 1.4 | 0.213 |
| Preoperative T5-T12 kyphosis (°) | | 1.0 | 0.8 to 1.1 | <0.001 |
| Postoperative T5-T12 kyphosis (°)^1^ | | 3.5 | -3.9 to 10.9 | 0.355 |
| Postoperative T5-T12 kyphosis across etiology (°)^1^ | |  |  |  |
|  | Idiopathic | Reference | Reference | Reference |
|  | Congenital | 0.3 | -7.7 to 8.4 | 0.932 |
|  | Neuromuscular | -2.5 | -9.6 to 4.6 | 0.487 |
| T5-T12 kyphosis change during slope 1 (°)^1,2^ | | 22.2 | 7.1 to 37.4 | 0.004 |
| T5-T12 kyphosis change during slope 2 (°)^1,2^ | | -12.4 | -51.1 to 26.3 | 0.529 |
| T5-T12 kyphosis change during slope 1 across etiology (°)^1,2^ | |  |  |  |
|  | Idiopathic | Reference | Reference | Reference |
|  | Congenital | -8.9 | -26.1 to 8.4 | 0.312 |
|  | Neuromuscular | -17.9 | -34.0 to -1.8 | 0.030 |
| T5-T12 kyphosis change during slope 2 across etiology (°)^1,2^ | |  |  |  |
|  | Idiopathic | Reference | Reference | Reference |
|  | Congenital | 11.1 | -28.5 to 50.7 | 0.581 |
|  | Neuromuscular | 11.9 | -27.8 to 51.6 | 0.556 |
| The graph shows the mean line per etiology (based on a male with the median preoperative T5-T12 kyphosis) and the individual patient lines. For clarity, the individual lines are shown as straight lines between only the first and last postoperative follow-up. For the statistical analysis, all follow-up points in between were also considered. Using the above predictors (gender, preoperative T5-T12 kyphosis and etiology), the follow-up T5-T12 kyphosis can be predicted for patients undergoing SDS treatment. The marginal R2 value of the above model was 0.81, outlining that the above predictors could predict 81% of the variation between data points.  ^1^The initial change is added for all groups, but for the congenital and neuromuscular group, additional changes are later added. Since the idiopathic group is the reference group, and no changes are added for the idiopathic group, the initial changes are identical to changes in the idiopathic group.  ^2^Since the changes over time were best described with a non-linear spline curve (with the inflection point after 3.5 years), differences in slopes are shown separately for the first (slope 1) and second (slope 2) part of the spline curve. | | | | |
