# Supplementary material for: Efficacy of the spring distraction system for different etiologies of early onset scoliosis: evaluating an evolving treatment concept
Source: Spine Deform. 2026 Jun 2;14(5):1921–33. doi: 10.1007/s43390-026-01368-0 (PMC13553710; doi:10.1007/s43390-026-01368-0)
Supplement: Supplementary file 3 [file 43390_2026_1368_MOESM4_ESM.docx]

| **Appendix 4. Linear mixed model results of L1-S1 lordosis over time** | | | | |
| --- | --- | --- | --- | --- |
| **Predictors** | | **Estimates** | **95% CI** | ***p* value** |
| Female | | -4.1 | -10.9 to 2.7 | 0.230 |
| Preoperative L1-S1 lordosis (°) | | 0.6 | 0.3 to 0.8 | <0.001 |
| Postoperative L1-S1 lordosis (°)^1^ | | 27.0 | 13.3 to 40.7 | <0.001 |
| Postoperative L1-S1 lordosis across etiology (°)^1^ | |  |  |  |
|  | Idiopathic | Reference | Reference | Reference |
|  | Congenital | 0.9 | -11.1 to 12.9 | 0.882 |
|  | Neuromuscular | -12.7 | -23.4 to -2.0 | 0.021 |
| L1-S1 lordosis change per year (°)^1^ | | 2.5 | -0.3 to 5.3 | 0.084 |
| L1-S1 lordosis change per year across etiology (°)^1^ | |  |  |  |
|  | Idiopathic | Reference | Reference | Reference |
|  | Congenital | 0.0 | -3.1 to 3.2 | 0.980 |
|  | Neuromuscular | -1.0 | -4.1 to 2.0 | 0.505 |
| The graph shows the mean line per etiology (based on a male with the median preoperative L1-S1 lordosis) and the individual patient lines. For clarity, the individual lines are shown as straight lines between only the first and last postoperative follow-up. For the statistical analysis, all follow-up points in between were also considered. Using the above predictors (gender, preoperative L1-S1 lordosis and etiology), the follow-up L1-S1 lordosis can be predicted for patients undergoing SDS treatment. The marginal R2 value of the above model was 0.55, outlining that the above predictors could predict 55% of the variation between data points.  ^1^The initial change is added for all groups, but for the congenital and neuromuscular group, additional changes are later added. Since the idiopathic group is the reference group, and no changes are added for the idiopathic group, the initial changes are identical to changes in the idiopathic group. | | | | |
