# Supplementary material for: Efficacy of the spring distraction system for different etiologies of early onset scoliosis: evaluating an evolving treatment concept
Source: Spine Deform. 2026 Jun 2;14(5):1921–33. doi: 10.1007/s43390-026-01368-0 (PMC13553710; doi:10.1007/s43390-026-01368-0)
Supplement: Supplementary file 4 [file 43390_2026_1368_MOESM5_ESM.docx]

| **Appendix 5. Patient complications and UPROR data** | | | | | | | | | | | |
| --- | --- | --- | --- | --- | --- | --- | --- | --- | --- | --- | --- |
| **Patient** | **Etiology** | **Event** | **Time from index surgery to event (months)** | **Clavien-Dindo-Sink classification** | **Complication type** | | | | | **Treatment** | **UPROR** |
|  |  |  |  |  | **Disease** | | **Surgery** | | **Implant** |  |  |
| **P1** | C | End of spring | 16 | - |  | |  | |  | Re-tension surgery | 1 |
|  |  | Rod fracture | 60 | 3 |  | |  | | X | Fix later at PSF | 0 |
| **P2** | C | Dura tear | 0 | 2 |  | | X | |  | Bed rest | 0 |
|  |  | End of spring | 11 | - |  | |  | |  | Re-tension surgery | 1 |
| **P3** | C | Failure connector | 43 | 3 |  | |  | | X | PSF ≥12 years | 0 |
| **P4** | I |  |  |  |  | |  | |  |  |  |
| **P5** | NM | Connector failure | 18 | 3 |  | |  | | X | PSF ≥12 years | 0 |
| **P6** | C |  |  |  |  | |  | |  |  |  |
| **P7** | NM | Excessive kyphosis | 7 | 3 |  | |  | | X | Lengthening of gliding mechanism | 1 |
|  |  | Failure distal anchor | 18 | 3 |  | |  | | X | Replacement of distal anchor | 1 |
|  |  | End of spring | 44 | - |  | |  | |  | PSF <12 years | 1 |
| **P8** | I | Excessive implant kyphosis | 19 | 3 |  | |  | | X | PSF ≥12 years | 0 |
| **P9** | NM | End of spring | 29 | - |  | |  | |  | Re-tension surgery | 1 |
| **P10** | NM | Deep SSI | 2 | 3 |  | | X | |  | I&D + antibiotics | 1 |
|  |  | Rod protrusion | 8 | 3 |  | |  | | X | Rod bending and repositioning | 1 |
|  |  | Failure distal anchor | 10 | 3 |  | |  | | X | Replacement of distal anchor | 1 |
| **P11** | NM | Rod fracture | 29 | 3 |  | |  | | X | Conversion to 5.5 mm rods | 1 |
|  |  | Failure rod cross-connector + rod slippage | 49 | 3 |  | |  | | X | PSF <12 years | 1 |
| **P12** | NM | End of spring | 34 | - |  | |  | |  | Re-tension surgery | 1 |
|  |  | Pulmonary insufficiency | 49 | 3 | X | |  | |  | Admission + antibiotics | 0 |
| **P13** | NM | Rod slippage in connector | 26 | 3 |  | |  | | X | Connector + set screw replacement + spring re-tensioning | 1 |
| **P14** | C | End of spring | 21 | - |  | |  | |  | Re-tension surgery | 1 |
|  |  | Excessive implant kyphosis | 42 | 3 |  | |  | | X | Conversion to 5.5 mm rods + stacking two connectors | 1 |
| **P15** | C |  |  |  |  | |  | |  |  |  |
| **P16** | NM | Excessive implant kyphosis | 7 | 3 |  | |  | | X | Adding 1 more proximal level to the distal anchor + adding rod cross connector | 1 |
| **P17** | NM | Pulmonary insufficiency | 0 | 4 | X | |  | |  | ICU admission | 0 |
|  |  | End of spring | 5 | - |  | |  | |  | Re-tension surgery | 1 |
| **P18** | I | Rod fracture | 22 | 3 |  | |  | | X | Fix later at PSF | 0 |
|  |  | Rod fracture | 40 | 3 |  | |  | | X | PSF ≥12 years | 0 |
| **P19** | NM | Rod protrusion | 1 | 3 |  | |  | | X | Distalisation of connector + rod bending | 1 |
|  |  | Rod fracture | 34 | 3 |  | |  | | X | Fix later at PSF | 0 |
|  |  | Failure connector | 37 | 3 |  | |  | | X | PSF <12 years | 1 |
| **P20** | NM | Excessive implant kyphosis | 10 | 3 |  | |  | | X |  | 0 |
|  |  | Failure anchor | 17 | 3 |  | |  | | X | Anchor replacement + stacking two connectors | 1 |
|  |  | Buttress slippage | 32 | 2 |  | |  | | X | Expectative | 0 |
| **P21** | C | Deep SSI | 18 | 3 |  | | X | |  | I&D + 3 months of antibiotics | 1 |
|  |  | Adding on | 69 | 3 |  | | X | |  | PSF ≥12 years | 0 |
| **P22** | NM | End of spring | 38 | - |  | |  | |  | Re-tension surgery | 1 |
| **P23** | NM | Death (respiratory insufficiency) | 25 | 5 | X | |  | |  |  | 0 |
| **P24** | NM | End of spring | 15 | - |  | |  | |  | Re-tension surgery | 1 |
|  |  | Rod disconnection | 74 | 3 |  | |  | | X | PSF ≥12 years | 0 |
| **P25** | NM |  |  |  |  | |  | |  |  |  |
| **P26** | NM | Wound dehiscence | 1 | 3 |  | | X | |  | I&D | 1 |
|  |  | Rod slippage | 3 | 3 |  | |  | | X | Distal anchor replacement | 1 |
|  |  | Rod fracture | 23 | 3 |  | |  | | X | Fix later at PSF | 0 |
|  |  | Rod fracture | 26 | 3 |  | |  | | X | PSF ≥12 years | 0 |
| **P27** | NM | Deep SSI | 1 | 3 |  | | X | |  | I&D + 3 months of antibiotics | 1 |
|  |  | Pulmonary insufficiency | 1 | 4 | X | |  | |  | ICU admission | 0 |
|  |  | Adding on | 37 | 3 |  | | X | |  | Conversion to 5.5 mm rods + including additional distal levels | 1 |
| **P28** | I | Rod fracture | 27 | 3 |  | |  | | X | Fix later at PSF | 0 |
| **P29** | C | Temporary NM loss | 0 | 3 |  | | X | |  | Staged procedure | 1 |
|  |  | Rod fracture | 19 | 3 |  | |  | | X | Conversion to 5.5 mm rods | 1 |
|  |  | Rod fracture | 32 | 3 |  | |  | | X | PSF ≥12 years | 0 |
| **P30** | I |  |  |  |  | |  | |  |  |  |
| **P31** | C |  |  |  |  | |  | |  |  |  |
| **P32** | NM |  |  |  |  | |  | |  |  |  |
| **P33** | NM | End of spring | 19 | - |  | |  | |  | Re-tension surgery | 1 |
|  |  | Death (respiratory insufficiency) | 36 | 5 | X | |  | |  |  | 0 |
| **P34** | NM |  |  |  |  | |  | |  |  |  |
| **P35** | I | Rod fracture | 21 | 3 |  | |  | | X | Conversion to 5.5 mm rods | 1 |
|  |  | Rod slippage | 36 | 3 |  | |  | | X | Additional apical screw fixation | 1 |
| **P36** | I |  |  |  |  | |  | |  |  |  |
| **P37** | NM | Excessive implant kyphosis | 44 | 3 |  | |  | | X | Conversion to 5.5 mm rods + stacking two connectors | 1 |
| **P38** | I | Failure connector | 44 | 3 |  |  | | X | | PSF ≥12 years | 0 |
| **P39** | NM | Excessive implant kyphosis | 15 | 3 |  |  | | X | | Stacking two connectors | 1 |
| **P40** | NM |  |  |  |  |  | |  | |  |  |
| **P41** | C | Malposition rod | 0 | 2 |  |  | | X | | Wait and see | 0 |
|  |  | Adding on | 20 | 3 |  | X | |  | | Including more proximal levels + stacking two connectors | 1 |
| **P42** | I | Rod slippage | 26 | 3 |  |  | | X | | PSF ≥12 years | 0 |
| **P43** | NM | Failure distal anchor | 23 | 3 |  |  | | X | | Wait and see | 0 |
| **P44** | C |  |  |  |  |  | |  | |  |  |
| **P45** | C | Deep SSI | 0 | 3 |  | X | |  | | I&D + 3 months of antibiotics | 1 |
|  |  | Pull-out proximal anchor | 3 | 3 |  |  | | X | | Changing to larger diameter screws + additional of proximal level. Temporarily fixing connector to aid in anchor ingrowth. After 4 months, additional surgery to dynamize the construct. | 2 |
|  |  | Rod fracture | 39 | 3 |  |  | | X | | Conversion to 5.5 mm rods | 1 |
| **P46** | NM |  |  |  |  |  | |  | |  |  |
| **P47** | C |  |  |  |  |  | |  | |  |  |
| **P48** | NM |  |  |  |  |  | |  | |  |  |
| **P49** | C |  |  |  |  |  | |  | |  |  |
| **P50** | NM |  |  |  |  |  | |  | |  |  |
| **P51** | NM | Failure distal anchor | 16 | 3 |  |  | | X | | Additional distal level + screw replacements + spring retensioning | 1 |
|  |  | Rod fracture | 40 | 3 |  |  | | X | | PSF <12 years | 1 |
| **P52** | NM | End of spring | 33 | - |  |  | |  | | Re-tension surgery | 1 |
| **P53** | NM |  |  |  |  |  | |  | |  |  |
| **P54** | NM | Disconnection rod | 37 | 3 |  |  | | X | | Fix later at PSF | 0 |
|  |  | Rod fracture | 50 | 3 |  |  | | X | | PSF ≥12 years | 0 |
| **P55** | NM |  |  |  |  |  | |  | |  |  |
| **P56** | NM |  |  |  |  |  | |  | |  |  |
| **P57** | NM |  |  |  |  |  | |  | |  |  |
| **P58** | NM |  |  |  |  |  | |  | |  |  |
| **P59** | NM |  |  |  |  |  | |  | |  |  |
| **P60** | NM |  |  |  |  |  | |  | |  |  |
| **P61** | NM |  |  |  |  |  | |  | |  |  |
| **P62** | NM | Pulmonary insufficiency | 0 | 4 | X |  | |  | | Admission ICU + intubation | 0 |
|  |  | Pulmonary insufficiency | 34 | 3 | X |  | |  | | Admission + antibiotics | 0 |
| **P63** | NM |  |  |  |  |  | |  | |  |  |
| **P64** | NM |  |  |  |  |  | |  | |  |  |
